# 1953-2023. Seventy Years of the Nerve Growth Factor: A Potential Novel Treatment in Neurological Diseases?

**DOI:** 10.14336/AD.2024.0573

**Published:** 2025-07-15

**Authors:** Davide Norata, Fioravante Capone, Francesco Motolese, Massimo Marano, Mariagrazia Rossi, Rosalinda Calandrelli, Marta Sacchetti, Flavio Mantelli, Vincenzo Di Lazzaro, Fabio Pilato

**Affiliations:** ^1^Department of Medicine and Surgery, Unit of Neurology, Neurophysiology, Neurobiology and Psychiatry, Università Campus Bio-Medico di Roma, Via Alvaro del Portillo, 21 - 00128 Roma, Italy.; ^2^Fondazione Policlinico Universitario Campus Bio-Medico, Via Alvaro del Portillo, 200 - 00128 Rome, Italy.; ^3^Radiology and Neuroradiology Unit, Department of Imaging, Radiation Therapy and Hematology, Università Cattolica del Sacro Cuore, Fondazione Policlinico Universitario Agostino Gemelli, Istituto di Ricovero e Cura a Carattere Scientifico (IRCCS), Rome, Italy.; ^4^Dompé Farmaceutici S.p.A., Via Campo di Pile -67100 L’Aquila, Italy.

**Keywords:** NGF, Nerve Growth Factor, Neurotrophins, Neuromodulation, Neurological disorders

## Abstract

Rita Levi-Montalcini's 1953 discovery of nerve growth factor (NGF) in mouse sarcoma tumors marked a groundbreaking moment in neuroscience. NGF, a key signaling molecule, became the first identified neurotrophic factor, influencing the growth, differentiation, and survival of neurons in both peripheral and central nervous systems. NGF and related neurotrophic factors hold therapeutic potential for various neurological disorders, such as Alzheimer's Disease, Parkinson's Disease, Huntington's Disease, amyotrophic lateral sclerosis, spinal cord injuries, neuropathies, traumatic brain injuries, and stroke. However, despite promising *in vitro* studies and animal models findings, NGF efficacy in patients remains unproven. Indeed, its use as a therapeutic agent faces challenges in delivery and clinical translation. This review delves into these challenges, exploring ongoing research on refined delivery methods, dosages, and safety profiles. Innovative strategies, including molecular mimicking, combination therapies, gene therapy, and coupling with neuromodulation techniques like transcranial magnetic stimulation and vagal nerve stimulation, are discussed. Incorporating nerve growth factor (NGF) into a comprehensive strategy may prove beneficial, particularly in non-neurodegenerative conditions such as stroke, trauma, and neuropathies. In these instances, NGF holds promise for promoting tissue regeneration and repair. Challenges persist in addressing the complexity of neurodegenerative pathologies for a combined therapeutic approach.

## INTRODUCTION

Rita Levi-Montalcini made a significant observation while studying the nervous system while working in Viktor Hamburger's laboratory at Washington University in St. Louis in 1953. She found that a soluble extract from a mouse sarcoma tumor induced remarkable axon growth in explanted sympathetic ganglia, which are clusters of nerve cells. This discovery marked the identification of the nerve growth factor (NGF), a crucial signaling molecule that plays a key role in the development, maintenance, and survival of nerve cells [1].

NGF was the first discovered member of the family of neurotrophic factors, signaling proteins that promote the growth, differentiation, and survival of neurons. Levi-Montalcini's work laid the foundation for a vast body of scientific research on NGF and other neurotrophic factors, leading to a deeper understanding of the mechanisms underlying neural development and function. The discovery of the NGF was a groundbreaking finding in the field of neuroscience. In 1986, Rita Levi-Montalcini was awarded the Nobel Prize in Physiology or Medicine for her pivotal role in the discovery of NGF, sharing the honor with Stanley Cohen, her collaborator in identifying and characterizing NGF. This recognition highlighted the importance of their work in unraveling the mysteries of how nerve cells develop, interact and survive. The study of NGF and neurotrophic factors continues to be an exciting area of research, enabling researchers to understand various neurological disorders and potential therapeutic interventions.

NGF was the first identified member of the neurotrophins, a family of soluble neurotrophic proteins that includes brain-derived neurotrophic factor (BDNF), neurotrophin 3 (NT3), and neurotrophin 4/5 (NT4/5) [2].

NGF is synthesized as a precursor molecule (proNGF) which is then processed into its mature form. This processing occurs either through cleavage by furin and/or pro-protein convertases within the trans-Golgi network or by extracellular plasmin and/or other metalloproteases. The result is a mature, non-covalently linked, homodimer with a molecular weight of approximately 26 kDa, known as mNGF or simply NGF [3, 4]. Both proNGF and NGF are biologically active and play a crucial role in the growth, development, and maintenance of neurons in both peripheral and central nervous system (PNS and CNS, respectively). They achieve these functions by binding to specific cell-surface receptors [5]. Both proNGF and NGF have the capability to attach to a specific tyrosine kinase receptor (tropomyosin kinase receptor A, TrkA, or Ntrk1) and to the pan neurotrophin receptor p75 (LNGFR or p75^NTR^), which can interact with all neurotrophins. Particularly, NGF shows higher affinity for binding TrkA and proNGF for p75^NTR^ [4, 6]. In the early 21st century, the "double play" role of p75NTR was extensively studied and understood. When p75NTR is part of a neurotrophin-Trk complex, it increases the binding affinity of the complex, enhancing its survival-promoting activity. However, p75NTR can also bind to neurotrophins independently of Trk (with assistance from another receptor called sortilin) and, in doing so, it promotes apoptotic mechanisms [6–11].

The identification of neurotrophic factors opened new possibilities for exploring their therapeutic potential in the treatment of various injuries and diseases affecting the central and peripheral nervous system, and the retina. These molecules have been investigated for their ability to promote the survival and growth of neurons, and retinal ganglion and photoreceptor cells. The application of neurotrophic factor-based therapies has been proposed for several conditions, including:

1. Degenerative diseases of the retina - Neurotrophic factors have been studied for their potential to support the survival of retinal ganglion cells and photoreceptors, offering potential treatments for degenerative eye diseases like retinitis pigmentosa, glaucoma and macular degeneration [12].
2. Alzheimer's Disease (AD) - The neuroprotective effects of neurotrophic factors have raised interest in their potential application for conditions involving neurodegeneration, such as AD [13].
3. Parkinson's Disease (PD) - Researchers have investigated the use of neurotrophic factors to support the survival of dopaminergic neurons in the brain, which are affected in PD [14].
4. Huntington's Disease (HD) - Neurotrophic factors have been explored for their potential neuroprotective effect on striatal Medium Spiny Neurons (MSN) and on cortico-striatal projections, which are primarily affected in HD [15].
5. Amyotrophic Lateral Sclerosis (ALS) - ALS, a motor neuron disease, prompted research into the use of neurotrophic factors to support the survival of motor neurons [16].
6. Spinal Cord Injury - Neurotrophic factors have been examined for their ability to promote nerve regeneration and functional recovery following spinal cord injuries [17].
7. Traumatic Brain Injury (TBI) - The potential neuroprotective effects of neurotrophic factors have been explored in the context of traumatic brain injuries [18].
8. Stroke - Studies have investigated the use of neurotrophic factors to enhance neuronal survival and recovery in the aftermath of stroke in animal models [19].

While the therapeutic potential of neurotrophic factors, including NGF, has been extensively explored, there are indeed some limitations to their use for therapeutic purposes. These may include challenges related to the delivery of neurotrophic factors to specific target areas, potential side effects, and the need for further understanding of the complex mechanisms involved. Additionally, the translation of promising preclinical findings into effective clinical treatments has proven to be a complex process, and ongoing research aims to address these challenges and refine the use of neurotrophic factors in therapeutic strategies for various neurological conditions.

The objective of this review is to provide a comprehensive summary of the most authoritative literature on the use of NGF as a potential therapeutic and prognostic element in neurological disorders along with exploring prospects in this field.

This review also looks into the evolving landscape of NGF-based therapies, including ongoing clinical trials and innovative research directions. Additionally, this review considers the potential combination therapies and interdisciplinary approaches that may shape the future of NGF utilization in neurological disorders, by addressing challenges related to clinical trial design, clinically relevant endpoints, delivery methods, dosages, and side effects.

## NGF IN NEUROLOGICAL DISORDERS

**Figure 1.**
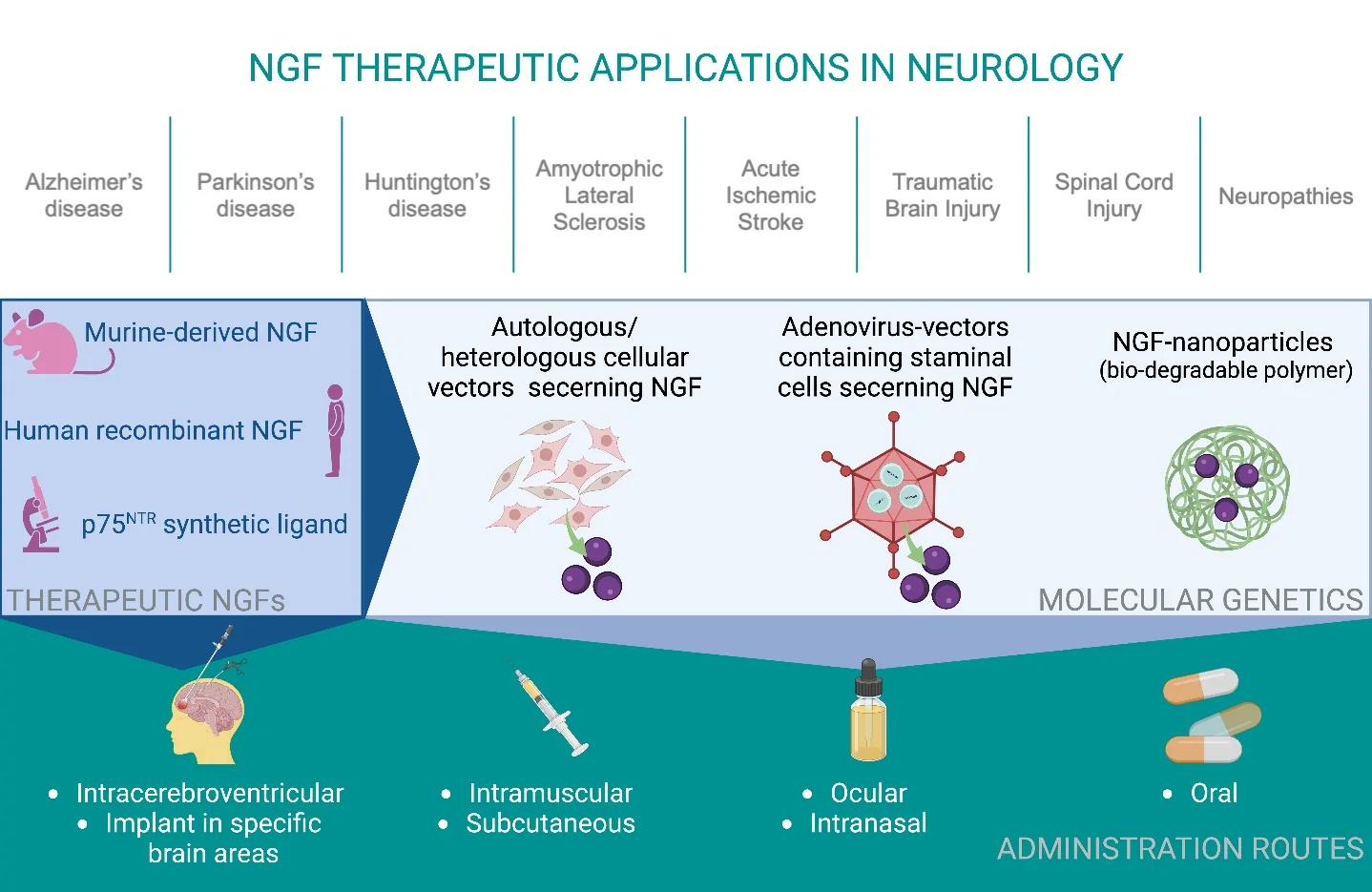
Schematic representation of potential therapeutic applications of nerve growth factor (NGF) in neurological disorders.

The therapeutic promise of NGF and other neurotrophic factors in addressing neurological diseases and conditions is widely acknowledged, underscoring their potential impact. While the clinical applications are currently in the experimental phase, the compelling prospect of leveraging these factors for effective treatments emphasizes their significant potential in the realm of neurology. This review specifically addresses data related to prototypical neurological diseases, as a significant portion of existing literature focuses on these. Our targets will be neurodegenerative disorders, including Alzheimer's disease, Parkinson's disease, Huntington’s disease, and Amyotrophic Lateral Sclerosis as well as stroke, and traumatic brain injury (Fig. 1).

The evidence from human studies is summarized in Table 1.

### Alzheimer's Disease

AD is the most common form of dementia in the elderly [20]. It is primarily characterized by the extracellular accumulation of beta-amyloid (Aβ) peptide in the brain, forming amyloid plaques, and the presence of intracellular tangles (neurofibrillary aggregates) mainly composed of hyperphosphorylated tau protein (p-tau) [21, 22]. The primary clinical manifestation of AD is a progressive decline in cognitive function, often accompanied by behavioral disturbances [23].

In the late 1970s and early 1980s, while investigating cognitive impairments in AD, multiple research teams established a connection between the depletion of cholinergic neurons in the basal forebrain and concurrent cognitive decline, forming the basis of the cholinergic hypothesis [24]. This hypothesis underlies modern treatments with cholinesterase inhibitors (ChEIs), such as donepezil, rivastigmine, and galantamine.

Cortically-projecting cholinergic neurons arise from the nucleus basalis of Meynert/*substantia innominata* (nMB/SI) and constitute a major component of forebrain circuitry mediating cognitive, specifically attentional processes and capacities [25, 26]. These neurons of basal forebrain cholinergic nuclei (BFCN) express both TrkA and p75^NTR^ NGF-receptors. Therefore, when NGF interacts with BFCN-neurons, it triggers a signaling cascade involving PI3K/Akt, MEK/ERK, and PLCγ pathways, which is crucial for neuronal survival. Dysfunction in this signaling pathway can lead to impaired cytoskeleton function, contributing to the severity of the cognitive symptoms in AD and accelerating generalized, telencephalic neurodegeneration [27-30].

**Table 1:** NGF-based therapies for Neurological Diseases in human beings.

*Neurological Disorder*	*References, study type*	*Tested molecule*	*Delivery Route*	*Efficacy results*	*Safety results*
*AD*	Olson et al., 1992 [38] Case report	Mouse NGF	Intracerebroventricular administration	Increase of cortical blood flow and brain nicotine uptake. Improvement of verbal episodic memory	Weight loss
	Eriksdotter-Jönhagen et al., 1998 [39] Phase I RCT	Mouse NGF	Intracerebroventricular administration	No significative cognitive improvement, changes in EEG, increased nicotine binding in several brain areas	Weight loss and back pain.
	Tuszynski et al., 2005 [40] Phase I RCT	rhNGF	Gene therapy (in autologous grafted fibroblasts)	Improvement in the rate of cognitive decline. Significant increases in cortical FDG-uptake after treatment (brain PET)	No long-term adverse events in 6/8 patients
	Rafii et al., 2014 [44] Phase I RCT	rhNGF	Bilateral stereotactic administration of an adeno-virus vector to the nucleus basalis of Meynert	No significative cognitive improvement, no decreases of FDG uptake in brain PET after 2 years, increased post-mortem brain NGF expression	No long-term adverse events
	NCT00876863 Phase II RCT	rhNGF	Bilateral stereotactic implatntation of an adeno-virus vector of NGF to the nucleus basalis of Meynert	NA	NA
	Wahlberg et al., 2012 [41]; Eriksdotter-Jönhagen et al., 2012 [42] Phase Ib, open label, study on 6 patients	rhNGF	Bilateral stereotactic implatntation of a encapsulated cell biodelivery (ECB) device containing human retinal cells with NGF to the nucleus basalis of Meynert	No significant cognitive improvement	Implantation and removal of devices were safe and well tolerated.
	NCT03069014 Phase II RCT	LM11A-31 (p75 ligand)	Oral administration	NA	NA
*PD*	Olson et al., 1991 [60] Case report	Mouse NGF (support for adrenal medula graft)	Intraputaminal administration	Prolonged effect of adrenal chromaffin grafts	Not reported
*ALS*	Li J-T et al., 2022 [16] Observational, retrospective study	Mouse NGF	Intramuscular injection	No significant differences in ALS progression	No long-term adverse events
*Ischemic stroke*	NCT03686163 Phase IV RCT	NGF	Intranasal	NA	NA
*TBI*	NCT01212679 Phase II RCT	NGF	Intranasal	NA	NA
	Chiaretti et al., 2008 [102] Case series	Mouse NGF	Intracerebroventricular administration	Improvement in EEG and SPECT parameters. An increase of doublecortin in CSF	Not reported
*Diabetic polyneuropathy*	Apfel et al., 1998 [143] Phase II RCT	rhNGF	Subcutaneous	Significant improvement of neuropathic symptoms after 6 months of treatment.	Dose-dependent hyperalgesia at the injection site.
	Apfel et al., 2000 [145] Phase III RCT	rhNGF	Subcutaneous	Not significant effect	Dose-dependent hyperalgesia at the injection site.
*Bell's Palsy*	Su et al., 2015 [154] Meta-analysis of 8 RCT	Mouse NGF / NGF (not specified)	Intramuscular injection	Favorable effects of NGF on clinical and neurophysiological responses	Not reported

AD, Alzheimer’s Disease; PD, Parkinson’s Disease; ALS, Amyotrophic Lateral Sclerosis; TBI, Traumatic Brain Injury; NGF, Nerve Growth Factor; rhNGF, recombinant human NGF; RCT, Randomized Controlled Trial; NA, not available.

Animal studies show that higher NGF levels are associated with improved cognitive functions [31-33], while studies on patients with mild cognitive impairment indicate that this condition is linked to reduced TrkA expression and higher proNGF levels [34]. Furthermore, matrix metalloproteinase 9 (MMP9) is upregulated, leading to the degradation of mature NGF. This process may contribute to the imbalance favoring a pro-apoptotic trigger by proNGF [5, 35].

Aberrant neuronal loss occurs in pathological aging, while normal aging involves a gradual loss of cholinergic function due to degeneration in dendrites, synapses, axons, and reduced trophic support. Imbalances in NGF and proNGF expression, TrkA and p75^NTR^ receptors, as well as changes in acetylcholine-related factors, may contribute to cholinergic dysfunction. Additionally, Aβ peptides’ deposition may worsen cholinergic dysfunction by interfering with NGF signaling and promoting tau hyperphosphorylation [36].Therefore, it is worth considering NGF and proNGF levels as potential biomarkers to assess the functional status of the cholinergic system, shedding light on the aging process in the brain, whether it's healthy or pathological [13].

The first proposal for therapeutic use of a neurotrophin was made in the 1980s, with NGF being the sole known member at the time. In an established animal model of age-related memory impairments, the intraventricular administration of NGF not only reversed memory deficits but also mitigated degenerative changes in the cholinergic neurons of the basal forebrain [37].

However, evidence in AD patients was less encouraging. A case report and a study on three patients, both involving the intraventricular injection of mouse-derived NGF, failed to demonstrate any cognitive improvement but highlighted the emergence of side effects such as back pain and excessive weight loss [38, 39].

Some phase I clinical trials and an open-label study have used gene therapy procedures to inject AD patients with human-derived NGF, genetically engineered using human vectors (autologous [40] or heterologous [41, 42]) or derived from adenoviruses [43, 44]. These trials have shown an absence of NGF-related adverse events but a questionable benefit. Cognitive outcome improvements (i.e., slowing the rate of cognitive decline) were observed only in some patients, thus with low statistical power.

Since the beginning of 2000s, various research teams have been investigating the therapeutic potential of multiple compounds in altering or inhibiting the expression of the p75^NTR^ receptor [45]. Some of these compounds include LM11A-31 [46], CATDIKGAEC [47, 48], and sulforaphane [49], tested both *in vitro* and *in vivo*. Additionally, other scientists have explored the effectiveness of administering the extracellular domain of p75^NTR^ to counteract the AD phenotype [50, 51].

Given the promising results in studies conducted on murine models, some of these compounds are currently undergoing clinical trials to understand the biochemical changes they may induce. One of these compounds (LM11A-31) is also the subject of a Phase II clinical trial involving patients with mild to moderate AD (ClinicalTrials.gov accession number NCT03069014; Available online: https://clinicaltrials.gov/ct2/show/NCT03069014?term=LM11A-31-BHS&draw=2&rank=1), however, as of November 2023, the results have not been published (for an extensive review on the topic see Bruno et al., 2023 [13]).

### Parkinson's Disease

PD is the second most prevalent neurodegenerative condition after Alzheimer's disease [52]. It is characterized by the gradual loss of dopaminergic neurons in the *substantia nigra pars compacta* (SNc) and the development of Lewy bodies (LBs) [52], intracellular inclusions abundant in α-synuclein, a significant pathological feature of PD and various other neurodegenerative disorders [53].

PD patients exhibit key motor deficits, including bradykinesia, rigidity, postural instability, and resting tremor [52]. They also experience various non-motor symptoms, such as depression, sensory abnormalities, sleep disturbances, and cognitive impairment [54, 55]. Impairments in controlling voluntary movements in PD patients arise from alterations in the functional organization of the basal ganglia, including the deficiency of dopaminergic neurotransmission in the striatum due to the loss of dopaminergic neurons in the SNc[56].

PD-animal models have indicated that repeated administration of NGF-dipeptide improved behavioral outcomes and exhibited neuroprotective effects against neuronal death caused by mitochondrial toxins such as 1-methyl-4-phenyl-1,2,3,6-tetrahydropyridine (MPTP) [57-59]. Therefore, NGF has been explored as a potential treatment for PD patients to promote the survival of dopaminergic neurons. However, challenges related to delivering NGF to the brain and concerns about potential side effects have posed significant obstacles. In 1991, a 23-day intraputaminal infusion of mouse-derived mNGF was attempted in a single patient with PD who had undergone an autologous graft of adrenal medulla into the putamen [60]. The rationale behind using mNGF was its supportive role for the engrafted adrenal medullary cells in the basal ganglia of PD patients [14, 61]. During the 13-month follow-up, the patient exhibited a rapid decrease in rigidity and hyperkinesia, consistent with previous studies on autologous grafts of chromaffin tissue in PD patients [62, 63]. The specific effect of mNGF support for the graft was identified in a gradual improvement of motor functions, extending for 11 months post-graft procedure. Thus, NGF treatment showed potential in extending the effects of adrenal chromaffin grafts in human PD [60].

Beyond its therapeutic role, NGF and proNGF levels might also serve as biomarkers for treatment response in PD patients, considering that levodopa administration may contribute to increased NGF plasma concentrations, which are typically reduced in PD patients [64]. Furthermore, another neurotrophin, the BDNF, is currently employed as a biomarker in studies focusing on neuroprotection and physical exercise in PD, hinting at the extensive role that these molecules can play in this field [65].

### Huntington’s Disease

Huntington's disease (HD) is a neurodegenerative disorder mainly characterized by motor symptoms (including chorea, dystonia, and impaired voluntary movements), cognitive decline, and psychiatric disturbances. It is caused by an autosomal dominant expansion of a trinucleotide repeat (CAG) in the exon 1 of the huntigtin gene (HTT), leading to the production of an abnormal form of the huntingtin protein [66]. This mutant HTT (mHTT) accumulates in neurons, causing neuronal dysfunction and eventual cell death, particularly in the basal ganglia and cerebral cortex [67, 68]. The cortico-striatal-thalamo-cortical circuits are notably affected, with significant striatal atrophy observed in humans [69]. Caudate volume decline begins 5-15 years prior to diagnosis, representing a defining characteristic of symptomatic HD [70].

Studies using brain MRI of living patients or post-mortem brain images support the observation that striatal damage increases progressively in HD patients before symptom onset, in direct correlation with the length of the CAG repeat [71, 72]. Therefore, striatal volume consistently serves as a discriminating marker between individuals with and without the HTT mutation, and it also tracks the disease progression [73, 74]. Moreover, HD patients present a notable loss of GABAergic medium spiny neurons (MSNs) in the caudate-putamen, along with a neuronal decline in the globus pallidus, accompanied by both cortical gray and white matter loss [75, 76].

The use of NGF as a diagnostic marker to indicate the severity of the disease and even a therapy is supported by evidence showing lower NGF levels in HD patients compared to healthy individuals [77].

Traditional animal models of HD involve the injection of toxins such as Quinolinic acid (QA) and the mitochondrial toxin 3-nitropropionic acid (3-NP), which lead to striatal lesions that predominantly destroy GABAergic MSNs while preserving cholinergic neurons, akin to what occurs in HD patients [78, 79].

Experiments exploring therapeutic effects of NGF in HD models have shown that there is a variation in results depending on the mode of NGF administration. Studies during which NGF was infused into the striatum before or simultaneously with QA injections showed protection limited to cholinergic interneurons and a specific increase in cholinergic markers, while markers for GABAergic MSNs remained unaffected [80-82]. Consequently, NGF infusion does not seem to be a viable therapeutic approach for HD. The cellular delivery of NGF (via retrovirally transfected fibroblasts, rodent progenitor cells, or GFAP-responsive stem cells) effectively preserves both cholinergic and noncholinergic neurons from degeneration caused by excitotoxicity or mitochondrial dysfunction [83-86]. This protective effect extends to GABAergic MSNs, suggesting that cellular delivery of NGF could safeguard susceptible populations of striatal neurons in HD patients [87].

### Amyotrophic Lateral Sclerosis

ALS, an aggressively advancing and ultimately fatal motor neuron disorder, was among the earliest candidates for neurotrophin-based therapeutic approaches. This disease is characterized by the degeneration of both upper and lower motor neurons, culminating in a devastating paralysis that invariably leads to death [88].

The role of neurotrophin signaling pathways in spinal motor neuron development and maintenance is complex [89]. Studies have examined NGF expression in both in postmortem tissues of ALS patients and in mouse models. Some findings suggest an increased NGF expression in the spinal cord of Sod1G93A mice, specifically in astrocytes, which may contribute to motor neuron degeneration [90-92]. However, results from postmortem spinal cord tissue analysis in ALS patients are conflicting, with reports of both an increased and decreased NGF expression [92-94]. There have been no reports of direct assessments of NGF's role in ALS pathogenesis using blocking antibodies or genetic deletions in ALS models.

The hypothesis has been that NGF may act in a pro-degenerative manner via the p75^NTR^, as motor neurons do not typically express TrkA [95-97]. However, the postmortem analysis of spinal cords from ALS patients suggested motor neurons might increase TrkA receptor expression, shifting neurotrophin dependence from BDNF to NGF [93]. This led to a clinical trial using intramuscular mouse-derived NGF injections for ALS treatment, involving 28 days of daily injections followed by 6 months without treatment, with the goal of providing neuroprotection and promoting the survival of motor neurons. Despite the well-tolerated treatment, there was no significant change in ALS progression in the treatment group [16].

NGF-based therapies for ALS are still experimental, but they are one of many alternative avenues being explored in the search for effective potential treatments for this devastating disease.

### NGF in Traumatic Brain Injury and Acute Ischemic Stroke

NGF has been tried in several neurodegenerative diseases, but its effects have been also evaluated in other neurological conditions.

Traumatic Brain Injury (TBI) is a severe head trauma resulting from an external force that disrupts normal brain function, affecting about 69 million individuals globally each year [98]. Current treatment options target mainly symptom relief. Research in alternative strategies is ongoing, including the use of neuroprotective growth factors.

Ischemic stroke is an acute cerebrovascular event that can result in significant brain damage due to the interruption of blood supply to the brain, leading to metabolic changes, cell damage and death.

Therefore, brain damage for an ischemic stroke is differs from TBI in terms of mechanism of disease, but common features may be found such as inflammation, increased extracellular calcium levels, excitotoxicity, impairment of the blood-brain barrier and free-radical mediated toxicity [99, 100]. In this perspective, growth factors like NGF have been explored to study their neuroprotective and regenerative therapeutic potentials in stroke and TBI.

Preclinical studies and case series in children with TBI showed NGF’s safety and efficacy in rescuing neurons and improving motor function [18, 101, 102]. Similar positive outcomes were observed in animal models of ischemic stroke [19, 103].

Recent clinical trials investigated intranasal NGF delivery in patients with TBI (Phase II, NCT01212679, 2017), or with ischemic stroke (Phase IV, NCT03686163, 2020), but results have not been reported yet.

### NGF in Spinal Cord Diseases, Peripheral Neuropathies, and Related Disorders

#### Spinal Cord Injury

Spinal Cord Injury (SCI) triggers an increased expression of NGF mRNA in both the spinal cord and dorsal root neurons (DRG) [104-109]. However, the overexpression of NGF elicits contradictory effects in SCI. While it may promote spinal cord repair and the survival of both spinal and DRG neurons [17, 110, 111], NGF-related mechanisms are also associated with significant clinical symptoms of SCI, such as neuropathic pain and urinary incontinence.

#### Neuropathic Pain

Neuropathic pain following SCI is a debilitating condition, causing sleep disturbances and depression, with a prevalence ranging between 38-70% among the SCI population [112-114].

Voltage-gated sodium channels (VGSCs) play pivotal roles in pain sensation and conduction, serving as potential targets for pain management and a possible link between neuropathic pain and NGF may be found. Following a spinal cord injury, mice exhibit abnormal expression of VGSC Nav1.7 in neurons within both the deep and superficial *laminae* layers of the spinal dorsal horn, as well as an upregulated expression in the DRG. Notably, these sites also demonstrate overexpression of NGF. Based on this evidence, it has been suggested that the aberrant expressions of VGSC Nav1.7 and NGF likely share a common pathway [108, 115].

Therapies capable of penetrating the blood-brain barrier and inhibiting NGF or its related signaling pathways (including Nav1.7 blockers) have shown efficacy in alleviating neuropathic pain induced by SCI in animal models, but also a delayed spinal cord repair [108, 116-119]. Therefore, the role of pro- or anti-NGF treatment in neuropathic pain is still highly debated, especially in the presence of nerve damage, where NGF treatment could theoretically restore normal nerve function.

#### Urinary Incontinence

One of the most promising applications of NGF-related treatments in SCI therapy is the management of urinary incontinence. NGF expressed in the bladder and conveyed via bladder afferent pathways has been demonstrated to be linked with the induction of detrusor overactivity (DO) and heightened C-fiber afferent excitability post-SCI [120-122].

Research has demonstrated that chronic NGF administration into the lumbosacral spinal cord or the bladder triggered DO and elevated the firing rate of dissociated capsaicin-sensitive bladder afferent neurons [123-125]. Additionally, immunoneutralization of NGF in the lumbosacral spinal cord post-SCI reduced DO and detrusor sphincter dyssynergia in rats [126].

Recent studies have also revealed that treatments with systemic anti-NGF antibody or with specific mitogen-activated protein kinases (MAPK) inhibitors (MAPK/ERK cascade is frequently triggered by NGF) lead to a decrease in urinary frequency and in the number of non-voiding bladder contractions (NVCs) in SCI mice [127, 128].

### Neuropathies and Related Disorders

As per the conventional neurotrophic model, NGF is synthesized and released within target tissues, and subsequently captured by specific receptors expressed on sensory and sympathetic nerve terminals. It is then transported retrogradely to the neuronal cell body, where it plays a crucial role in the survival and maintenance of these neurons [129, 130]. Thus, any disturbance in NGF synthesis, transport, and utilization in peripheral neurons can give rise to the nerve dysfunction characteristic of peripheral neuropathies [131-134]. This underscores the etiological significance of NGF in the development of neuropathic symptoms linked to conditions such as diabetes, HIV infections, or chemotherapy. It also highlights the potential of this neurotrophin as a pharmacological tool in the treatment of peripheral and cranial neuropathies.

#### Diabetic Peripheral Neuropathy

Diabetic neuropathy is a highly prevalent condition that substantially affects patients by increasing falls, causing pain and reducing quality of life [135]. It includes a spectrum of clinical symptoms resulting from damage to the peripheral and autonomic nervous systems, collectively referred to as various forms of neuropathy [136]. The duration of diabetes and levels of hemoglobin A1c (HbA1c) are the major predictors of diabetic neuropathy [137]. Some studies revealed a correlation between reduced NGF serum levels in diabetic patients and the clinical manifestation of peripheral neuropathy [138, 139].

Recombinant human NGF (rhNGF) was initially tested in a Phase I trial on healthy subjects after encouraging results in animal models [133, 140] and, displayed moderate side effects like myalgias and injection site hyperalgesia [141, 142]. A subsequent Phase II trial with 250 diabetic polyneuropathy patients demonstrated improved neuropathic symptoms with rhNGF treatment, but also highlighted side effects, limiting its therapeutic usefulness [143, 144]. Unfortunately, a Phase III RCT with 1019 patients showed similar side effects but failed to show significant benefits from NGF treatment [145].

A recent clinical trial investigating Tocotrienol-Rich Vitamin E (Tocovid) supplementation in diabetic neuropathy patients indicated significantly elevated serum NGF levels after eight weeks of Tocovid supplementation compared to the placebo group. This finding suggests a potential association between vitamin E metabolism and the NGF pathway in these disorders [146].

#### Guillain-Barré Syndrome

Guillain-Barré syndrome (GBS) is an autoimmune disease affecting the peripheral nervous system, causing muscle weakness, hyporeflexia or areflexia, and in severe cases, complete paralysis. It is the most common cause of acute or subacute flaccid weakness globally, after the eradication of poliomyelitis [147, 148].

While no direct link between NGF and GBS exists, some research has explored the potential role of neurotrophic factors in both the development and recovery from GBS. Animal models, mainly rodents, have been used to study the therapeutic potential of NGF and other neurotrophic factors in experimental autoimmune neuritis (EAN). This disease shares similar pathological characteristics with human idiopathic demyelinating polyradiculoneuritis, the most common variant of GBS.

Schwann cells in rats with EAN exhibit increased p75^NTR^ gene expression, indicating an enhanced affinity for NGF. This suggests a potential role in promoting the regeneration of damaged axons [149].

Another intriguing connection between the NGF pathway and GBS involves monosialoganglioside GM1. GBS and its variants are associated with antibodies against specific gangliosides. GM1 enhances TrkA autophosphorylation, modulating NGF-TrkA signaling to promote neurite outgrowth in specific sympathetic nerve cell lines [150-152]. In neural cell lines derived from GBS patients with IgG anti-GM1 positivity, these antibodies blocked GM1’s positive effect on NGF-TrkA signaling, thereby interfering with NGF-induced neurite outgrowth and regeneration [153].

#### Bell’s Palsy

Idiopathic facial nerve paralysis (IFNP), commonly known as Bell's palsy, is a sudden, unexplained, and unilateral paralysis of the face [147].

A systematic review and meta-analysis from 2014, which encompassed eight clinical trials, critically assessed the impact of NGF treatment on Bell's palsy [154]. The findings indicated favorable effects of NGF on response rates, assessed both clinically and neurophysiologically through compound muscle action potential amplitude. Although the authors acknowledged that the limited number and quality of trials hindered the ability to draw definitive conclusions, the therapeutic potential of NGF in this condition remains notably high [154].

## CHALLENGES AND PERSPECTIVES

### Delivery Challenges

Delivery into the target tissues is a primary challenge in the use of NGF as a therapeutic agent, particularly to the brain and spinal cord. This difficulty arises from the presence of the blood-brain barrier (or the blood-nerve barrier for the peripheral nervous system), that represent a significant impediment limiting the entry of large molecules like NGF and the other neurotrophins into the central (and peripheral) nervous system [155-157]. Furthermore, when administered peripherally, NGF is prone to rapid catabolism. As a result and for many years, the most common delivery method has been the invasive direct NGF injection into the brain, via intra-cerebro-ventricular administration, a technique associated with significant adverse events [158], with the exception of a few studies employing ineffectively intramuscular NGF administration [16, 38, 39].

Other administration routes attempted in animal models were intranasal delivery and topical administration of NGF on the ocular surface, both capable of non-invasively and safely transporting therapeutic concentrations of NGF to selected brain areas [159-161]. Studies on animal models of AD demonstrated that both intranasal and ocular administration of human-derived NGF preserve the neurotrophic potential of native NGF without triggering pain-related responses [161, 162]. Although intranasal administration has not been used within clinical trials, it has recently been successfully employed in a child with acquired traumatic brain injury and in an infant suffering from severe neurological impairment due to late-onset Streptococcus agalactiae meningitis [101, 163]. More recently, the same Authors extended their experience with intranasal NGF by treating a small cohort of children in vegetative state after out-of-hospital cardiac arrest, and children with post-traumatic unresponsive wakefulness syndrome[164, 165]. The results of these preliminary experiences with intranasal NGF delivery in humans are promising and pave the way for further research on potential clinical applications of NGF for central nervous system indications.

Ocular administration of NGF is currently approved for the treatment of neurotrophic keratopathy [12], but is has also been explored in some clinical trials for the treatment of ocular conditions such as glaucoma and retinitis pigmentosa. These trials did not conclusively demonstrate significant benefits of the treatment, but did not yield evidence of systemic adverse events [166-169].

It is important to emphasize that studies on peripheral neuropathies have frequently used subcutaneous administration of NGF, with contrasting results [144].

The advent of gene therapy and endogenous NGF expression technology has revolutionized the landscape by offering more promising modalities of NGF delivery. For instance, in the treatment of AD, genetically engineered NGF has been delivered via autologous or heterologous human vectors, as well as vectors derived from animals or adenoviruses. Although its therapeutic effectiveness remains uncertain, a good safety profile with a lack of adverse effects has been demonstrated [40-44].

Another innovative delivery method tested *in vitro* and animal models, involves encapsulating NGF within a bio-degradable polymer coating to form NGF nanoparticles (e.g., NGF polyethylene-glycol poly-lactic-co-glycolic acid nanoparticles - NGF-PE-PLGA-NPs). This approach enables the direct transport of NGF to target cells [170-172]. While this model has not yet been applied in humans, its prospects are promising.

An interesting consideration regarding delivery routes is that the delivery route also impacts the neural networks influenced by the administered NGF. In patients with Huntington's Disease, intra-striatal NGF administration is neurotrophic solely for cholinergic neurons, while transport facilitated by genetically engineered cells has shown improvements in both cholinergic and GABAergic networks. Thus, the transport with genetically engineered cells is potentially more effective than the intra-stratial delivery, particularly in a condition where the loss of GABAergic neurons is a crucial pathogenetic event [87].

For a comprehensive review of NGF delivery paradigms, see Alastra et al., 2021 [173].

### Safety considerations

NGF administered, especially at high doses or its prolonged exposure, can induce side effects, including inflammation and pain, often through its nerve-sensitizing role [174], thus a balance between its therapeutic benefits and potential adverse effects is crucial for its clinical applications.

Intramuscular administration of NGF led to back pain and excessive weight loss [38, 39], while intra-cerebro-ventricular administration was linked to neurosurgical complications [158].

The use of topical methods (intranasal and ocular) significantly reduce the occurrence of adverse events, limiting them to the topical application site, such as mild and transient conjunctival hyperemia, mild periocular pain, and photophobia following ocular administration [166-169].

Gene therapy to deliver the NGF gene directly to the affected nervous system area demonstrated its safety and efficacy in animal studies [40-44].

### Perspectives

While NGF-based therapies can be potentially beneficial, they are still in the experimental stage, with some trials failing to demonstrate certain NGF therapeutic effects due to delivery issues in the neurological disorders described above.

This section focuses on possible current, promising, and new perspectives in the neurological field.

Intense research is ongoing to refine delivery methods, dosages, and safety profiles. Researchers are exploring various approaches to enhance the delivery and effectiveness of NGF in treating degenerative diseases. Additionally, the exploration of gene therapy, molecular mimicking, and combination therapies, such as the integration of NGF with stem cell therapy or brain stimulation techniques, offers a multifaceted approach to unlocking the full potential of NGF in treating degenerative neurological diseases.

#### NGF Gene Therapy

NGF gene therapy is a promising approach for delivering NGF to the brain and spinal cord. It involves human cellular implanted vectors (autologous or heterologous) or adenoviruses, that are engineered to produce and secrete human or murine-derived NGF.

Preliminary data obtained in rodents and primates demonstrated its efficacy in enhancing cognitive and motor functions in models of AD, PD, and HD. Clinical trials are currently assessing the potential of NGF gene therapy for various neurological disorders, but the results have not been entirely convincing [40-44].

#### Molecular Mimicking

Recent studies have explored the development of chemical compounds that mimic the effects of NGF on neurons. These molecules are typically smaller than NGF, they can pass the blood-brain barrier to reach the brain and spinal cord and they are less capable of causing side effects than NGF itself [175].

Several small molecule drugs mimicking the effects of NGF are currently in preclinical development [13]. Many of these molecules act by altering or inhibiting the expression of p75^NTR^. Most have been studied in relation to Alzheimer's disease, both *in vitro* and *in vivo* [46-51], but clinical trials on patients are still ongoing (ClinicalTrials.gov accession number NCT03069014; Available online: https://clinicaltrials.gov/ct2/show/NCT03069014?term=LM11A-31-BHS&draw=2&rank=1).

One of the extensively studied molecules is GK-2, designed to mimic the β-turn sequences of loop 1 of the NGF molecule [176]. *In vitro* and animal model studies have demonstrated the neuroprotective effects of GK-2 in both AD and PD[57, 177]. GK-2 stimulated neurogenesis and synaptogenesis when administered peripherally in rat models of ischemia, resulting in a notable reduction of the infarct volume and improved neurological functions [178, 179]. Additionally, in an *in vivo* TBI model, GK-2 significantly enhanced limb motor function compared to untreated animals [180].

#### Combination Therapies

The effects of combining NGF with other therapeutic approaches to enhance overall treatment strategies have been investigated.

An intriguing model of these combined treatments is the association of NGF with Tacrolimus. Tacrolimus, an immunosuppressive agent widely used in organ transplantation, has demonstrated neural regeneration promotion [181]. Studies have illustrated synergistic effects of Tacrolimus and NGF combined treatment in regenerating neural structures and promoting functional recovery in rats following injuries to the sciatic nerve or spinal cord [182, 183].

Researchers are also exploring the use of NGF in combination with stem cell therapy to stimulate the growth of new neurons in the brain, particularly after stroke or in neurodegenerative disorders. *In vitro* and *in vivo* studies on rats have demonstrated that NGF can induce natural stem cells (NSCs) to differentiate into specific neurons. Combining NGF-nanoparticles with NSC transplantation has shown significant improvements in learning and memory functions in rats with Alzheimer’s disease, replenishment of basal forebrain cholinergic neurons, and mitigation of neuronal death following experimental ischemic stroke [172, 184-186].

Another combination therapy introduced hypoxia-responsive elements into an adenovirus vector containing transplanted NSCs. This induced the NSCs to express NGF specifically in hypoxic sites, thereby promoting the recovery of spinal cord injuries in rats [187].

These innovative applications of NGF may overcome the limitations of current NGF therapies and pave the way for more effective and safer treatments for neurological diseases. The combined approach may offer several advantages, with therapeutic effects acting on complementary pathways to produce an increased overall effect.

## COUPLING NGF AND NEUROMODULATION TECHNIQUES

Neuromodulation techniques have been developed to influence or regulate neural network activity within the nervous system. Nowadays, various non-invasive brain stimulation (NIBS) techniques have been approved for the treatment of disorders ranging from chronic pain to movement disorders and psychiatric conditions [188]. Evidence suggests that neuromodulation has a role as therapeutic option in neuropsychiatric disorders but might also be a tool for promoting neuroprotection - e.g., in the context of limiting the damage due to a stroke [189]. The effect of these techniques is evident not only from the neurophysiological perspective, but also in terms of alterations of cerebrospinal fluid [190] and plasma levels of key proteins involved in brain homeostasis (for a comprehensive review that serves as an example of this observation, regarding the TMS-related neurobiological changes in AD models, see Bashir et al., 2022 [191]).

**Figure 2.**
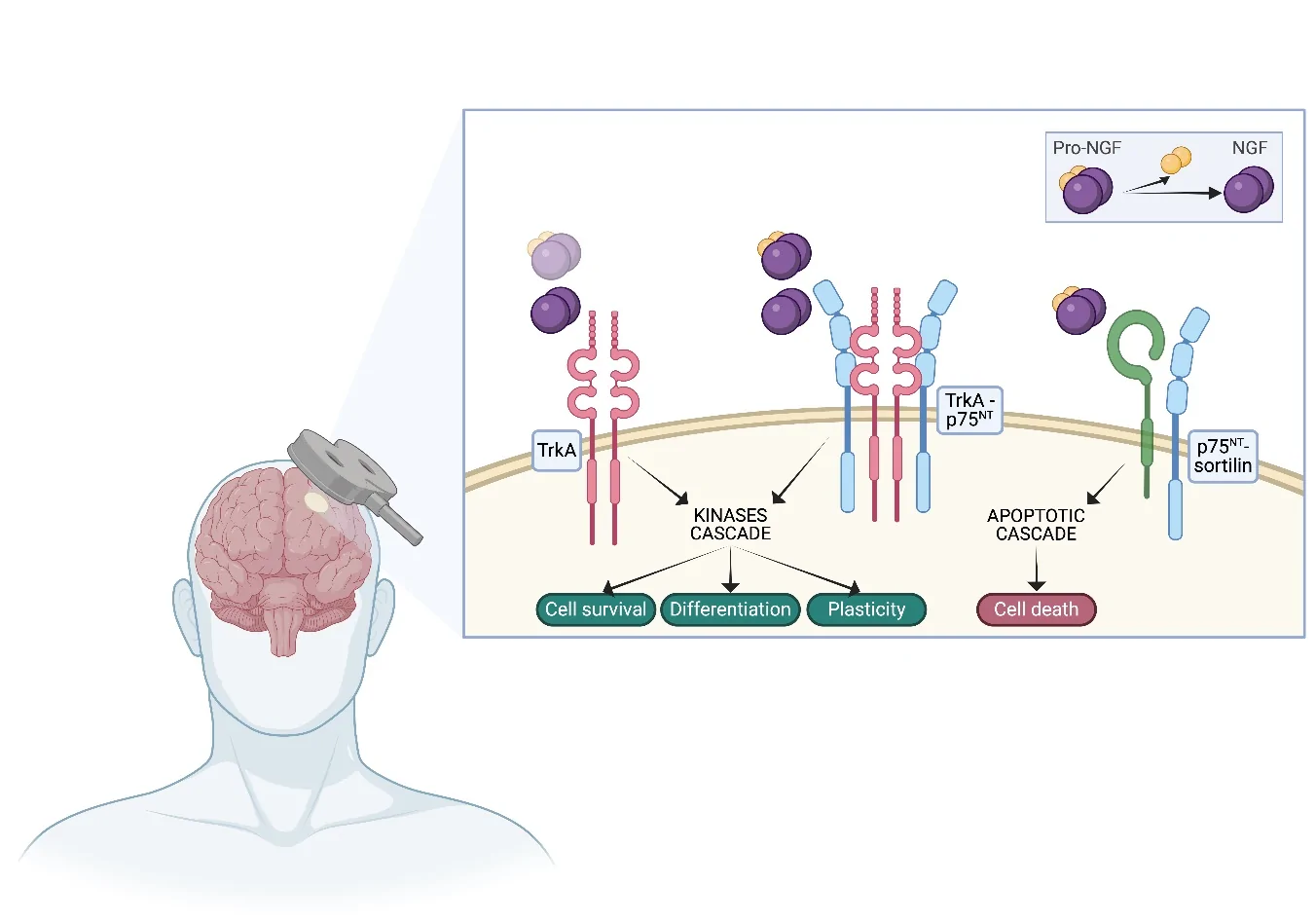
**Cellular transduction mechanisms mediated by the binding of nerve growth factor (NGF) to its receptors**. Non-invasive brain stimulation techniques (NIBS), such as the represented transcranial magnetic stimulation (TMS), are proposed to potentially intervene by modulating these mechanisms. In addition to enhancing the bioavailability of NGF and its precursor pro-NGF, specific NIBS techniques may promote the expression of tyrosine kinase receptors (Trk), which, through kinase cascade pathways, increase neuronal proliferation, differentiation, and synaptic plasticity.

Thus, it is not surprising that some studies have been focusing on the exploration of the potential synergies between NIBS techniques and neurotrophic factors. In fact, these techniques aim to modulate neural activity, enhance survival, and potentially elevate neurotrophins levels, contributing to therapeutic interventions in neurological diseases (Fig. 2).

Neuromodulation encompasses a diverse array of NIBS techniques, ranging from transcranial magnetic stimulation (TMS) - in which a magnetic field induces electric currents in specific regions of the brain - to transcranial electrical stimulation (tES), in which low-intensity electrical currents are applied to the scalp.

TMS is the most widely used NIBS technique. It uses brief and high-intensity magnetic fields to perturbate cortical excitability. Electric current flow through a coil held over the scalp, generating a magnetic field. This induces a current within the cerebral cortex that runs parallel but in the opposite direction to the coil's current and has the capability to influence cortical excitability.

When TMS is delivered as a train of pulses -the so-called repetitive TMS (rTMS)- the effects on cortical excitability persist for some time after the end of stimulation. This variably prolonged after-effect is thought to be the result of neural plasticity changes, such as long-term potentiation and long-term depression (LTP/LTD)-like mechanisms. LTP/LTD refer to the long-lasting increase or decrease of strength of synaptic connections among neurons, and is thought to be the basic mechanism of learning and memory [192]. In particular, low-frequency rTMS - i.e., 1-Hz - reduces cortical excitability while higher stimulation frequencies -i.e., > 5 Hz- increase it [193].

Several studies have reported beneficial effects of both low-frequency and high-frequency rTMS on up-regulating neurotrophin levels in in the blood, hippocampus, and cortex of healthy subjects and animals [194-200].

Some studies have focused on how rTMS could influence brain-derived neurotrophic factor (BDNF) levels in dementia. A study found increased BDNF levels in the basal forebrain and hippocampus in animal models of AD only after high-frequency (20 Hz) rTMS, even though both low- and high-frequency rTMS improved cognition [201].

In a study on humans, Velioglu and coworkers observed increased peripheral level of BDNF after two-week of high-frequency rTMS over the left lateral parietal cortex in AD patients [202]. RTMS has also demonstrated effectiveness in modulating NGF brain levels in AD mouse models [196, 203].

Much has been written on the potential application of rTMS for enhancing functional recovery after stroke.

Guo et al. found that rTMS improved cognition and reduced the sizes of cerebral lesions in an animal model of middle cerebral artery occlusion [204]. The authors observed the upregulation of BDNF and its TrkB receptor at the hippocampal level of the ischemic hemisphere, thus speculating that modulation of BDNF signaling pathway might be critical in functional recovery after stroke [204].

There are several studies that have shown that rTMS - both at high- and low-frequency, depending on the chosen site of stimulation - is beneficial for improving motor function rehabilitation after stroke. A study investigating the impact of a 2-week low-frequency (1 Hz) rTMS on ischemic stroke patients with upper limb hemiparesis revealed elevated serum levels of mature BDNF at the end of the treatment, indicating that this neurotrophic factor plays a role in the rTMS-related beneficial effects on motor function [205].

Finally, the effect of rTMS on neurotrophins level has been explored in individuals with ALS. However, available data are controversial, since the findings showed no significant fluctuations in BDNF blood values during rTMS protocols for ALS patients [197, 206, 207].

Transcranial direct current stimulation (tDCS) is a specific form of tES in which a low-intensity direct current is delivered at the scalp through two electrodes producing the increase of cortical excitability at the anode - or active electrode - and the decrease at the cathode [208]. Although the exact mechanisms of action of tDCS are unclear, research suggests that tDCS might induce neural plasticity phenomena, provide neuroprotective effects, stimulate the release of brain-derived neurotrophic factors, and mitigate neuronal apoptosis [209].

Regarding neural plasticity, a study on the application of electrical direct current to rat brain slices has shown the induction and modulation of the LTP phenomenon, with anodal direct current stimulation (DCS) enhancing it and cathodal DCS reducing it [210].

The effect of tDCS on LTP might occur through the induction of early genes, including zinc finger transcription factor binding protein clone 268 (zif268), also known as nerve growth factor-induced gene A (NGFI-A), as it can be induced by NGF itself. This suggests that the potential of tDCS to induce neuroplasticity changes might be influenced by NGF levels [211].

As briefly anticipated before, in a 2023 prospective case series, a combined treatment approach using intranasal human recombinant NGF (hr-NGF) and ten sessions (one session per day for 10 consecutive days) of 20-minute anodal tDCS to the left dorsolateral prefrontal cortex (DLPF) has been studied, and showed significantly improved functional, electrophysiological, and clinical outcomes in children in a chronic vegetative state following prolonged out-of-hospital cardiac arrest [164].

### Perspective in Neuromodulation Techniques

#### Focused Ultrasound (FUS)

A new frontier in neuromodulation is the use of ultrasound for therapeutic purposes in neurological disorders. Magnetic Resonance-guided Focused Ultrasound (MRgFUS) is a versatile tool approved for specific indications such as essential tremor or motor symptoms of Parkinson’s disease [212]. The application of high-intensity focused ultrasound (HIFU) raises the temperature on a defined focus, causing protein denaturation, DNA breakdown, and thermal coagulative necrosis [212]. This therapeutic thermal ablation applied through the intact skull, delivers energy from the cortex to deep structures, producing a lesion on a specific node of a networking, resulting in the desired therapeutic effect [212].

The ability of MRgFUS to transiently increase Blood-Brain Barrier (BBB) permeability through localized BBB tight junction opening is noteworthy [212]. Studies on patients with brain tumors demonstrated the safety and feasibility of MRgFUS for BBB opening, with the aim of enhancing the delivery of chemotherapeutic agent in the brain region of interest [213, 214].

This same approach might be applied for facilitating non-invasive delivery of other drugs in specific brain areas [215]. Applying FUS to open the BBB for enhancing neurotrophic factor delivery has proven efficacious in experimental PD models [216]. Combining FUS with intravenous neurotrophin infusion increased neuron fiber density in the caudate putamen in a mouse model of early stage PD, impacting dopamine release [217]. In another study, the synergistic use of FUS and brain-penetrating polymeric nanoparticles, delivering glial cell line derived neurotrophic factor (GDNF) in animal PD models, increased GDNF levels in the target region, providing lasting improvement of neural transmission in that area. Other studies showed improved of locomotor function with the combined administration of GDNF and FUS [218-221].

The intranasal administration of BDNF followed by FUS significantly enhanced BDNF distribution in treated regions in both human PD patients and animal models [222, 223].

#### Transcutaneous Vagal Nerve Stimulation (tVNS)

Nuclei of the Vagus Nerve have widespread connections with different parts of the nervous system including the basal forebrain (that contains areas that degenerate in the early stage of AD), the reticular formation (implied in the maintenance of the consciousness level) and numerous cortical sites. Therefore, the vagal nerve has been the object of several studies that aimed at modulating vagal activity. Currently, both implantable Vagal Nerve Stimulation (VNS) and non-invasive transcutaneous (tVNS) devices are used in clinical practice. VNS is approved as adjunctive treatment for pharmacologically resistant epilepsy and depression, while tVNS is approved for migraine and cluster headache [224]. Beyond these established clinical applications, both preclinical and clinical studies have highlighted other potential beneficial effects of vagal nerve stimulation, such as the anti-inflammatory effects or the promotion of neuroplasticity changes in the brain. This makes vagal nerve stimulation a potential treatment for various diseases ranging from stroke rehabilitation [225], TBI, neurodegenerative disorders such as Parkinson’s disease, where tVNS was able to engage key oscillatory activities improving motor and non-motor functions [226-228], and Alzheimer’s disease [229], to non-neurological conditions such as sepsis, lung injury, rheumatoid arthritis, obesity, and diabetes [224, 230]. Furthermore, VNS has also demonstrated anti-nociceptive effects, making it a viable option for pain control in conditions like fibromyalgia and migraine [224].

The presence of neurotrophin receptors has been confirmed in vagal nerve afferents and efferents, in the nodose ganglion, and other brain areas receiving vagal projections [231]. VNS induces changes in brain expression of NGF and BDNF, which are related to stimulation intensity and frequency. Since the latter parameters define which vagal fibers are recruited, it can be assumed that also neurotrophins expression depends on which vagal fibers or sensory afferents are activated, as other studies have shown [232, 233].

In conclusion, evidence suggests that vagal nerve stimulation might increase neurotrophic factors levels and their receptor expression in glial cells. This effect is likely due to the modulation of brain inflammatory responses and by interfering with the mechanism regulating the neurotrophic factors expression and maturation [233].

## Conclusion

Throughout seven decades, extensive research has elucidated the pivotal role of NGF in fundamental processes within developmental and adult neurobiology and NGF has been identified as a key factor influencing the survival, growth, and differentiation of nerve cells in both the peripheral and central nervous systems. Subsequent research has unveiled substantial beneficial properties inherent to NGF, particularly in the realms of cutaneous, ophthalmic, and neurological fields. In the area of neurological disorders, NGF has been scrutinized for its potential in both neurodegenerative and non-neurodegenerative conditions.

Although the administration of NGF in neurodegenerative disorders has demonstrated efficacy in *in vitro* studies and animal models, these effects have not been replicated yet in patients with neurodegenerative diseases. While animal models may be perceived as limiting due to their partial replication of the neurobiological changes that are characteristic of human pathology, literature data propose that a comprehensive approach, with NGF supplementation might be just one factor of a multifaceted strategy, that yields efficacy. Nevertheless, the complexity of neurodegenerative pathologies poses a formidable challenge even for a combined therapeutic approach. A more effective strategy might be a combined approach in non-neurodegenerative pathologies, such as ischemic stroke, brain or spinal trauma, and neuropathies. In these conditions, NGF exhibits the potential to stimulate function, regeneration and repair of damaged tissues, since surviving neurons retain full functionality and do not manifest impairment in their regenerative capabilities, which may be further augmented through the utilization of neurotrophins.
